# Supplementary material for: Modeling glioblastoma heterogeneity as a dynamic network of cell states
Source: Mol Syst Biol. 2021 Sep 16;17(9):e10105. doi: 10.15252/msb.202010105 (PMC8444284; doi:10.15252/msb.202010105)
Supplement: Supplementary file 6 — Source Data for Figure 5 [file MSB-17-e10105-s004.zip › Figure5A_sourcedata/GSEA_3071/hallmarks_stateVI.GseaPreranked.1623742557031/HALLMARK_PI3K_AKT_MTOR_SIGNALING.html]

Details for gene set HALLMARK\_PI3K\_AKT\_MTOR\_SIGNALING[GSEA]

|  || Dataset | state63071 |
| Phenotype | NoPhenotypeAvailable |
| Upregulated in class | na\_neg |
| GeneSet | HALLMARK\_PI3K\_AKT\_MTOR\_SIGNALING |
| Enrichment Score (ES) | -0.23594922 |
| Normalized Enrichment Score (NES) | -0.9445921 |
| Nominal p-value | 0.52418303 |
| FDR q-value | 0.69001293 |
| FWER p-Value | 1.0 |
Table: GSEA Results Summary

  

Fig 1: Enrichment plot: HALLMARK\_PI3K\_AKT\_MTOR\_SIGNALING      
 Profile of the Running ES Score & Positions of GeneSet Members on the Rank Ordered List

  

| PROBE | GENE SYMBOL | GENE\_TITLE | RANK IN GENE LIST | RANK METRIC SCORE | RUNNING ES | CORE ENRICHMENT || 1 | DDIT3 |  |  | 14 | 1.086 | 0.0563 | No |
| 2 | SQSTM1 |  |  | 147 | 0.667 | 0.0567 | No |
| 3 | MKNK2 |  |  | 326 | 0.524 | 0.0364 | No |
| 4 | PFN1 |  |  | 347 | 0.513 | 0.0593 | No |
| 5 | TRIB3 |  |  | 387 | 0.495 | 0.0759 | No |
| 6 | ACTR3 |  |  | 486 | 0.459 | 0.0741 | No |
| 7 | MAP2K3 |  |  | 550 | 0.440 | 0.0810 | No |
| 8 | RALB |  |  | 635 | 0.415 | 0.0807 | No |
| 9 | EGFR |  |  | 669 | 0.407 | 0.0941 | No |
| 10 | NFKBIB |  |  | 864 | 0.366 | 0.0606 | No |
| 11 | ARPC3 |  |  | 1139 | 0.325 | 0.0026 | No |
| 12 | ARHGDIA |  |  | 1153 | 0.323 | 0.0168 | No |
| 13 | UBE2D3 |  |  | 1361 | 0.296 | -0.0242 | No |
| 14 | AKT1S1 |  |  | 1363 | 0.296 | -0.0081 | No |
| 15 | PPP1CA |  |  | 1525 | 0.276 | -0.0374 | No |
| 16 | ECSIT |  |  | 1562 | 0.273 | -0.0323 | No |
| 17 | CAB39 |  |  | 1569 | 0.272 | -0.0189 | No |
| 18 | SLC2A1 |  |  | 1606 | 0.268 | -0.0140 | No |
| 19 | PDK1 |  |  | 1609 | 0.268 | 0.0003 | No |
| 20 | DUSP3 |  |  | 1743 | 0.256 | -0.0224 | No |
| 21 | PIN1 |  |  | 1924 | -0.268 | -0.0575 | No |
| 22 | NCK1 |  |  | 2174 | -0.309 | -0.1094 | No |
| 23 | CAMK4 |  |  | 2189 | -0.313 | -0.0960 | No |
| 24 | TIAM1 |  |  | 2303 | -0.334 | -0.1088 | No |
| 25 | STAT2 |  |  | 2345 | -0.342 | -0.1013 | No |
| 26 | ADCY2 |  |  | 2537 | -0.386 | -0.1329 | No |
| 27 | SMAD2 |  |  | 2594 | -0.399 | -0.1263 | No |
| 28 | CDK2 |  |  | 2746 | -0.439 | -0.1439 | No |
| 29 | PIK3R3 |  |  | 2903 | -0.495 | -0.1598 | No |
| 30 | E2F1 |  |  | 3140 | -0.613 | -0.1913 | No |
| 31 | CXCR4 |  |  | 3302 | -0.759 | -0.1938 | Yes |
| 32 | CDK1 |  |  | 3396 | -0.883 | -0.1707 | Yes |
| 33 | MAPK10 |  |  | 3398 | -0.886 | -0.1219 | Yes |
| 34 | CDKN1B |  |  | 3426 | -0.951 | -0.0766 | Yes |
| 35 | PLCB1 |  |  | 3468 | -1.045 | -0.0301 | Yes |
| 36 | ITPR2 |  |  | 3556 | -1.388 | 0.0228 | Yes |
Table: GSEA details [plain text format]

  

Fig 2: HALLMARK\_PI3K\_AKT\_MTOR\_SIGNALING: Random ES distribution      
 Gene set null distribution of ES for **HALLMARK\_PI3K\_AKT\_MTOR\_SIGNALING**
